# The Ebola epidemic is ongoing in West Africa and responses from China are positive

**DOI:** 10.1186/s40779-015-0031-8

**Published:** 2015-04-03

**Authors:** Jing-Min Zhao, Shi-Jun Dong, Jin Li, Jun-Sheng Ji

**Affiliations:** Department of Pathology and Hepatology, Beijing 302 Hospital, Beijing, 100039 China; Center of Medical Information, Beijing 302 Hospital, Beijing, 100039 China; Department of Infectious Diseases, Beijing 302 Hospital, Beijing, 100039 China

**Keywords:** Ebola virus, Ebola virus disease, Prevention and control

## Abstract

The ongoing Ebola outbreak poses an alarming risk to the countries of West Africa and beyond. On August 8, 2014, the World Health Organization (WHO) declared the cross-country Ebola outbreak a Public Emergency of International Concern. China has had no confirmed cases of Ebola. In this paper, virologic characteristics, pathogenesis, clinical manifestations, laboratory examination and prophylactic vaccines and therapeutic drugs of Ebola are summarized. Importantly, active responses and actions from China are introduced. Moreover, the key issues in the future prevention and control of Ebola were also addressed.

## Background

Ebola virus disease (EVD) is an acute severe illness which is often fatal if untreated. The origin of the virus is unknown, but fruit bats (Pteropodidae) and other non-human primates are considered as potential host of the Ebola virus (EBOV) [1,2]. EVD, initially discovered in 1976, was named after the Ebola River in Yambuku, Democratic Republic of Congo, where the first series of cases occurred. Previous EVD outbreaks were localized to remote areas in Central Africa, while the most recent epidemic in West Africa spread to rural and even urban areas. The EVD fatality rate reported in past outbreaks ranged from 25% to 90% [3].

The current Ebola outbreak in West Africa (first case reported on March 23, 2014) is the most serious and extensive outbreak since the EBOV was discovered. The number of incident cases and deaths was greater than other outbreaks in history. The most severely affected countries, including Guinea, Sierra Leone and Liberia have just survived from long periods of conflict and instability, and are equipped with fragile health systems which are short-handed in human and infrastructural resources. As of November 23, 2014, 15,935 Ebola cases and 5,689 deaths have been reported in eight affected countries, including Guinea, Liberia, Mali, Sierra Leone, Nigeria, Senegal, Spain and the United States of America. There are a total of 592 health-care workers (HCWs) infected with EVD as of November 23 and 340 of them have died [4].

Notably, evidence from the current Ebola epidemic and previous outbreaks strongly suggest the following epidemiological features of EBOV: non-airborne (not transmitted through a cough or sneeze) with a primary transmission route of direct contact with contaminated fluids, broken skin or mucous membranes. However, questions still remain concerning vomit and diarrheal fluids. Interestingly, the family of America’s first diagnosed Ebola patient did not acquire infection despite their common residence in which the patient had already been vomiting and having diarrhea. Due to lack of sufficient research, hard scientific evidence is still rare, so current assertions on the route of transmission are primarily based on observations by epidemiologists [5].

## Main text

### Virologic characteristics of EBOV

EBOV belongs to the family *Filoviridae*, a taxonomic group of enveloped, non-segmented, negative-strand RNA viruses with an 18.9 kb RNA genome. The *Filoviridae* virus family includes three genera: *Cueva virus*, *Marburg virus*, and EBOV. Ebola virions are uniform, with a diameter of 70–90 nm and a length of 300–1500 nm. The envelope, putatively derived from the host cell membrane, binds a 45–60 nm diameter nucleocapsid [6]. EBOV is composed of five different species: Bundibugyo, Sudan, Zaïre, Côte d’Ivoire and Reston. The first three species of EBOV have been linked to outbreaks in Africa, whereas the Côte d’Ivoire and Reston subspecies have not been associated with any Ebola hemorrhagic fever (EHF) human outbreaks to date. The virus responsible for the 2014 West African outbreak belongs to the Zaïre species. The genome of Zaïre EBOV encodes seven polyproteins and one non-structural protein, including the glycoprotein (GP), nucleoprotein (NP), RNA-dependent RNA polymerase (L), virion protein (VP) 24, VP30, VP35, VP40, and secreted glycoprotein (sGP) [7,8].

EBOV genome is relatively simple, but the molecular basis for EBOV interaction with host cells remains unclear. The mechanism by which EBOV infects cells might rely on two types of glycoprotein, in either a secreted or transmembrane form. Yang *et al*. [9] observed that sGP interacted with neutrophils through CD16b to inhibit early neutrophil activation, whereas the transmembrane glycoprotein interacted with endothelial cells likely contributing to the hemorrhagic symptoms of this disease. Additionally, the VP40 matrix protein and VP24 of EBOV might play a role with the formation of the surrounding envelope, virus assembly and budding [10,11], however, VP30 and VP35 might involve the transcription and replication of Ebola. NP, the primary structural protein, was associated with filovirus nucleocapsids [12]. Fortunately, to date, no evidence has shown that mutations could significantly alter the virulence, spread and pathogenicity of EBOV.

### Pathogenesis of EVD

The pathogenesis of EVD remains unclear. The incubation period of EBOV may depend on the infection route (e.g., 6 days for injection versus 10 days for contact). Migration of EBOV originates from the primary site of infection to regional lymph nodes, followed by vascular spread through the bloodstream to dendritic cells and macrophages within the liver, spleen, thymus, and other lymphoid tissues [13].

After infecting the host through mucosal membranes, broken skin, or through parental transmission, circulating EBOV can infect several cell types including monocytes, macrophages, dendritic cells, endothelial cells, hepatocytes and some epithelial cells. Although lymphocytes are known to be resistant against EBOV, they undergo apoptosis leading to a decrease in lymphocyte counts. An inference can be drawn that the cells above might express entry receptors of EBOV. Replication of filoviruses in widespread macrophages and dendritic cells, namely ‘sentinel’ cells, results in their necrosis and the release of abundant new viral particles into extracellular fluid [14]. Meanwhile, hepatocytes are destroyed and necrosis occurs, leading to dysregulation of clotting factors and even coagulopathy. Adrenocortical necrosis can also be observed, causing hypotension and impaired steroid synthesis. In addition to causing extensive tissue damage, EBOV also triggers macrophages, monocytes and other cells to release a cocktail of cytokines, chemokines, and a series of pro-inflammatory cytokines, such as tumor necrosis factor-alpha, interleukin (IL)-1beta, IL-6, macrophage chemotactic protein (MCP)-1, and nitric oxide, which not only destroy the vascular endothelium but also activate the coagulation cascade [15,16].

Interestingly, EBOV impairs the antigen-specific immune responses in both direct and indirect ways [17]. Filoviruses cause a severe and frequently fatal illness, partially, due to damage of dendritic cell function and promotion of lymphocyte apoptosis. As to the function of dendritic cells, they bear the primary responsibility to start the adaptive immune response and are a major site for replication of filoviruses. According to several *in vitro* investigation studies, infected dendritic cells fail to present antigens to naive lymphocytes attributing to the lack of mature cell development. These results suggest an underlying mechanism as to why patients dying from EVD may not develop antibodies to the virus [18,19]. Moreover, attrition of lymphocytes is another phenomenon observed in lethal EBOV infection. Although lymphocytes are not directly infected by EBOV, the overall systemic infection induces massive bystander apoptosis [20,21].

EBOV infection will result in multi-organ failure and shock, which appears to be caused by clotting disorders and vascular leakage. There are two stimulus factors, coagulation defects and virus-infected macrophages synthesizing cell-surface tissue factor (TF), which happen simultaneously and may contribute to the rapid progress and critical severity of the coagulopathy involved with EBOV infection. The former events appear to be triggered indirectly by the host inflammatory response, while the latter is activated the extrinsic coagulation pathway. Moreover, pro-inflammatory cytokines also induce macrophages to produce TF. In addition, other factors such as activated protein C and platelets may also be involved with the coagulation defects in EVD. A study by Hotchkiss RS and colleagues [22] showed that in EBOV-infected macaques, levels of activated protein C begin to decrease on day two post infection, while platelet counts decrease three or four days following infection, indicating that activated platelets are adhering to endothelial cells. Throughout EBV development, involvement of organ dysfunction such as hepatic injury will further cause a decline in plasma levels of certain coagulation factors.

### Clinical manifestations of EVD

The clinical symptoms of EVD are characterized with acuteness, severity and multi-organ involvement. The WHO’s definition of the incubation period or the time interval from infection with the virus to onset of symptoms, is 2 to 21 days [23]. The clinical presentations of EVD begins with an abrupt onset of fever and are characterized, initially, by a nonspecific flu-like illness such as fever, headache, malaise, myalgia, sore throat and gastrointestinal symptoms such as nausea vomiting, diarrhea and abdominal pain. Electrolyte derangements are mostly secondary to the diarrhea and vomiting. Severe hypokalemia along with moderate degrees of hypocalcaemia and hypomagnesaemia have been reported. In addition, capillary leakage combined with intravenous volume resuscitation commonly result in significant third spacing and peripheral edema. Although cough can occur, it is not a primary feature of this illness. Hiccups and dysphagia are often present, with severe associated discomfort. Hemorrhagic manifestations occurred in less than 10% of clinical cases, but these cases were relatively severe with poor outcomes. Primary causes of death are typically hypovolemic shock due to oro-gastric losses and third spacing (not hemorrhagic) related to severe electrolyte abnormalities. Renal failure is frequent in the late phase of severe disease. Prompt and accurate diagnosis of Ebola cases is essential to curb the Ebola outbreak. Differential diagnosis of EVD should take into account other acute Filovirus diseases as well as infectious diseases with similar clinical manifestations, such as malaria and typhoid fever.

### Laboratory examination of Ebola virus infection

Laboratory screening and diagnosis technologies of EBOV are limited, but are generally reliable. Early leukopenia, lymphopenia, atypical lymphocytosis and elevated AST and ALP are the main laboratory abnormalities indicative of EVD. Prothrombin and partial thromboplastin times are prolonged, and the detection of fibrin split products indicates disseminated intravascular coagulation. No single laboratory test will yield a definitive diagnosis of EBOV infection. The definitive diagnosis of EBOV infection can be made by laboratory tests including antibody-capture enzyme-linked immunosorbent assay (ELISA), antigen-capture detection tests, serum neutralization test, reverse transcriptase polymerase chain reaction assay (RT-PCR), electron microscopy, and virus isolation by cell culture (if necessary) [24]. Among these methods, RT-PCR is one of the most rapid and powerful tools for diagnosis of filovirus infection.

All laboratory testing should be performed in adherence with appropriate laboratory safety guidelines. Maximum biological containment conditions, such as biosafety level 4 laboratories (BSL-4), should be utilized for laboratory testing, regardless of whether the samples are inactivated. Any samples from patients, such as acute sera, postmortem tissue specimens, and materials collected during ecological investigations are of extreme biohazard risk. Therefore, maximum feasible precautions such as mask respirators, gloves and gowns are required when collecting any samples that may contain filoviruses. In September 2014, the WHO provided interim infection control recommendations addressing patient care, waste management, disposal of human remains, and other measures [25].

Chinese scientists have developed several detection kits for EBOV in August, including a fluorescence RT-PCR nucleic acid detection kit, colloidal gold immune chromatography antigen detection kit and ELISA antigen detection kit, which have been used in the laboratory examination of Ebola.

### Prophylactic vaccines and therapeutic drugs against EBOV

During the past several years, progress on development of potential vaccines against EBOV has been made, but the progress and development of antiviral drugs and other post-exposure interventions has been much slower. Retrieving the Cortellis database of Thomson Reuters combined with knowledge of EBOV to analyze current R&D of medicines and vaccines for EVD, the study findings show that there are mainly five important categories: vaccines, neutralizing antibodies, small-molecule anti-Ebola drugs, RNA interference (RNAi) drugs and nucleotide drugs. In each category, there are promising products under development that are worthy of attention.

Due to the lack of effective drugs available for EVD, the supportive treatments for complications such as septic shock, hematologic abnormalities, hypovolemia, refractory shock, hypoxia, electrolyte abnormalities, hemorrhage, multi-organ failure, and DIC should be emphasized. Severely ill patients require intensive supportive care [26]. For EVD patients, oral anti-emetics, anti-diarrheal therapy and adequate rehydration are very essential. In addition, infection prevention and control measures are a critical part of clinical management [27,28]. Although robust data are still lacking, clinical experience from EVD patients treated during the current outbreak suggests that the mortality rate associated with EVD can be significantly reduced through the provision of supportive care, and in particular critical care [29].

In the 2014 Ebola epidemic, after receiving an experimental drug called ZMapp, two American aid workers who fell sick with EVD in Liberia survived successfully. ZMapp was developed by San Diego-based private biotech firm Mapp Biopharmaceutical. A previous study suggests that ZMapp supplies the best option for experimental therapeutics used for treating EBOV-infected patients [30]. Potential treatments, including monoclonal antibody administration and small inhibitory RNA molecules, are still under scientific investigation.

To date, no vaccines have been licensed, but two potentially effective vaccines have entered the stage of human safety testing: cAd3-ZEBOV and rVSV-ZEBOV. The first vaccine is composed of a chimpanzee-derived adenovirus vector with an EBOV gene inserted. This vaccine is being jointly developed by GlaxoSmithKline with the US National Institute of Allergy and Infectious Diseases. An attenuated strain of Vesicular stomatitis virus (VSV), a pathogen normally found in livestock, is included in the second vaccine and one of the VSV genes is replaced by an EBOV gene. The second vaccine is being developed by the Public Health Agency of Canada in Winnipeg, while the license for its commercialization is held by NewLink Genetics Company, an American company in Ames, Iowa [31].

### Positive anti-Ebola response and action from China

Since the aftermath of the Severe Acute Respiratory Syndrome outbreak in 2003, China has developed a relatively strong system and accumulated precious experience for the prevention and control of emerging infectious diseases, which has been proven crucial and effective in controlling the H5N1 avian influenza epidemic in 2005, H1N1 influenza outbreak in 2009 and other public health emergencies. Facing the severe Ebola epidemic in West Africa, China has made positive and active actions, providing aids in the prevention and control of Ebola.

As of November 21, 2014, China successively provided four rounds of emergency aids with a total value of 750 million yuan to Ebola-affected countries. The medical staff from China has reached 400 man-times. In Sierra Leone, sample tests conducted by the mobile laboratory, supported by China, accounted for over 20% of total tests with accuracy up to 100%.Two-thirds of the international anti-Ebola material assistance received by Guinea came from China. Additionally, almost all the anti-Ebola materials within the ten countries surrounding the epidemic areas were offered by China. Approximately 1,000 people-composed of Chinese medical technicians and public health experts will be sent to the front-lines to battle against Ebola in the months ahead. An anti-Ebola training plan involving over 10,000 medical workers and community staff will soon be initiated by China.

China has implemented a serious of effective prevention and control strategies. For example, China has controlled the following entry gateways that Ebola has possibly invaded: 1) Entry gateway. China has instituted strict controls at airports, customs, and exit and entry ports, strengthened the quarantine work of each port and health monitoring of the passengers from Ebola epidemic areas. 2) Quarantine. Isolation and control of potential infection sources is the key measure to prevent and control Ebola. People returning from West African countries must remain in strict quarantine and people with positive or suspicious contact histories must remain in isolation. 3) National emergency. China has set up a strong infectious disease control and prevention system on the basis of knowledge acquired during the SARS outbreak following several public health urgencies. 4) Medical care. Medical staff is informed of the disease nature and an information bulletin is provided in a timely manner and strictly adhered to by the recommended infection control guidelines. 5) First aid. Once the Ebola virus infection appears, patients should be given symptomatic and supportive treatment. 6) Personal protection. Scientific understanding of the transmission route of Ebola can effectively control the spread of Ebola. 7) Etiquette custom. In the face of the highly contagious and lethal EBOV, we should change the handshake etiquette to avoid shaking hands and hugging with others. 8) Environmental sanitation. Maintaining proper sanitation is the key to prevent and control infectious diseases. Only when all these strategies have been implemented, will the transmission of disease in our country lessen.

In addition, Traditional Chinese Medicine has a place in preventing and treating emerging infectious diseases. In 2009 H1N1 epidemic, the Chinese medicine Maxingshigan-yinqiaosan formula showed the similar efficacy with oseltamivir in treating human H1N1. Traditional Chinese herbal therapy may be considered for a treatment option for Ebola [32].

## Conclusions

By analyzing detailed data from 3,343 confirmed and 667 probable Ebola cases collected in Guinea, Liberia, Nigeria, and Sierra Leone from March 23, 2014 to September 14, 2014, a WHO Ebola Response Team study shows that the basic reproduction number (R_0_), estimated current reproduction numbers (R) and corresponding doubling times for Guinea were 1.71, 1.81 and 15.7 days, respectively. Those values for Liberia were 1.83, 1.51 and 23.6 days, while for Sierra Leone they were 2.02, 1.38 and 30.2 days, respectively [33]. Notably, a susceptible-exposed-infectious-recovered model predicted the R_0_ of EVD in China is 1.53-3.54, which means a total of 6–194 imported cases [34]. The data above indicate that if strengthened control measures were not implemented, EVD cases and deaths associated with the disease would continue to grow in the near future in several currently affected areas and could even spread to surrounding countries.

Because the Ebola epidemic might last for another 6–9 months [35], developed countries and international aid groups should strengthen the support not only by sending more medical staff but also by helping these countries to establish suitable public health prevention and control systems. Positive anti-Ebola response and action plans from China have been proven effective.

Development of prophylactic vaccines and therapeutic drugs are the main focus in next step. These top international pharmaceutical companies should put the drive for the profit aside and join the global intelligence to develop protective vaccines and effective drugs. As discussed above, the epidemiological features, pathogenesis, prognosis and outcomes associated with Ebola remain unclear. Therefore, more research should be conducted on these aspects to provide evidence for the prevention and control of Ebola. Genetic mutation of EBOV should be closely monitored, especially the effect of mutations on the virulence and transmission routes. Hopefully, under the global effort, the challenge brought by Ebola outbreak will be conquered in the near future.

## Abbreviations

EBOV: Ebola virus
EHF: Ebola haemorrhagic fever
ELISA: Enzyme-linked immunosorbent assay
EVD: Ebola virus disease
GP: Glycoprotein
HCWs: Health-care workers
IL: Interleukin
MCP: Macrophage chemotactic protein
NP: Nucleoprotein
RNAi: RNA interference
sGP: secreted glycoprotein
TF: Tissue factor
VP: Virion protein
WHO: World Health Organization

## References

[1] Tosh PK, Sampathkumar P (2014). What clinicians should know about the Ebola outbreak. Mayo Clin Proc.

[2] Meyers L, Frawley T, Goss S, Kang C (2015). Ebola virus outbreak 2014: clinical review for emergency physicians. Ann Emerg Med.

[3] WHO: Ebola and Marburg virus disease epidemics: preparedness, alert, control, and evaluation[http://apps.who.int/iris/bitstream/10665/130160/1/WHO_HSE_PED_CED_2014.05_eng.pdf?ua=1]

[4] WHO: Ebola response roadmap-situation report [http://www.who.int/csr/disease/ebola/situation-reports/en/]

[5] Health Day: Airborne transmission of Ebola highly unlikely, experts say [http://consumer.healthday.com/infectious-disease-information-21/misc-infections-news-411/airborne-transmission-of-ebola-highly-unlikely-experts-say-693028.html]

[6] Huang Y, Xu L, Sun Y, Nabel GJ (2002). The assembly of Ebola virus nucleocapsid requires virion-associated proteins 35 and 24 and posttranslational modification of nucleoprotein. Mol Cell.

[7] Sanchez A, Kiley MP, Holloway BP, Auperin DD (1993). Sequence analysis of the Ebola virus genome: organization, genetic elements, and comparison with the genome of Marburg virus. Virus Res.

[8] Subbotina EL, Kachko AV, Chepurnov AA (2006). The properties of Ebola virus proteins. Vopr Virusol.

[9] Yang Z, Delgado R, Xu L, Todd RF, Nabel EG, Sanchez A (1998). Distinct cellular interactions of secreted and transmembrane Ebola virus glycoproteins. Science.

[10] Jasenosky LD, Neumann G, Lukashevich I, Kawaoka Y (2001). Ebola virus VP40-induced particle formation and association with the lipid bilayer. J Virol.

[11] Han Z, Boshra H, Sunyer JO, Zwiers SH, Paragas J, Harty RN (2003). Biochemical and functional characterization of the Ebola virus VP24 protein: implications for a role in virus assembly and budding. J Virol.

[12] Beer B, Kurth R, Bukreyev A (1999). Characteristics of Filoviridae: Marburg and Ebola viruses. Naturwissenschaften.

[13] Center for Diseases Control and Prevention: Ebola virus disease information for clinicians in U.S. healthcare settings//Ebola(Ebola virus diseases). http://www.cdc.gov/vhf/ebola/hcp/clinician-information-us-healthcare-settings.html. 2014-12-31.

[14] Bray M, Geisbert TW (2005). Ebola virus: the role of macrophages and dendritic cells in the pathogenesis of Ebola hemorrhagic fever. Int J Biochem Cell Biol.

[15] Villinger F, Rollin PE, Brar SS, Chikkala NF, Winter J, Sundstrom JB (1999). Markedly elevated levels of interferon (IFN)-gamma, IFN-alpha, interleukin (IL)-2, IL-10, and tumor necrosis factor-alpha associated with fatal Ebola virus infection. J Infect Dis.

[16] Hutchinson KL, Rollin PE (2007). Cytokine and chemokine expression in humans infected with Sudan Ebola virus. J Infect Dis.

[17] Sanchez A, Lukwiya M, Bausch D, Mahanty S, Sanchez AJ, Wagoner KD (2004). Analysis of human peripheral blood samples from fatal and nonfatal cases of Ebola (Sudan) hemorrhagic fever: cellular responses, virus load, and nitric oxide levels. J Virol.

[18] Geisbert TW, Hensley LE, Larsen T, Young HA, Reed DS, Geisbert JB (2003). Pathogenesis of Ebola hemorrhagic fever in cynomolgus macaques: evidence that dendritic cells are early and sustained targets of infection. Am J Pathol.

[19] Mahanty S, Hutchinson K, Agarwal S, McRae M, Rollin PE, Pulendran B (2003). Cutting edge: impairment of dendritic cells and adaptive immunity by Ebola and Lassa viruses. J Immunol.

[20] Hotchkiss RS, Coopersmith CM, Karl IE (2005). Prevention of lymphocyte apoptosis–a potential treatment of sepsis?. Clin Infect Dis.

[21] Parrino J, Hotchkiss RS, Bray M (2007). Prevention of immune cell apoptosis as potential therapeutic strategy for severe infections. Emerg Infect Dis.

[22] Hotchkiss RS, Karl IE (2003). The pathophysiology and treatment of sepsis. N Engl J Med.

[23] WHO: Ebola virus disease [http://www.who.int/mediacentre/factsheets/fs103/en/]

[24] Center for Diseases Control and Prevention. Diagnosis//Ebola (Ebola virus diseases). 2014. http://www.cdc.gov/vhf/ebola/diagnosis/.

[25] WHO: Interim infection prevention and control guidance for care of patients with suspected or confirmed Filovirus Haemorrhagic fever in health-care settings, with focus on Ebola [http://apps.who.int/iris/bitstream/10665/130596/1/WHO_HIS_SDS_2014.4_eng.pdf?ua=1&ua=1&ua=1] 10.1136/bmjph-2023-000556 PMC11251729 39015119

[26] CDC: Ebola Virus Disease Information for Clinicians in U.S. Healthcare Settings. [http://www.cdc.gov/vhf/ebola/hcp/clinician-information-us-healthcare-settings.html]

[27] Chertow DS, Kleine C, Edwards JK, Scaini R, Giuliani R, Sprecher A (2014). Ebola virus disease in West Africa–clinical manifestations and management. N Engl J Med.

[28] Canadian Critical Care Society, Canadian Assoc. of Emergency Physicians, Assoc. of Medical Microbiology & Infectious Diseases Canada: Ebola clinical care guidelines: A guide for clinicians in Canada. [http://cccsnew.businesscatalyst.com/website/Guidelines/Ebola%20Clinical%20Care%20Guidelines-2014-10-28.pdf]

[29] Kreuels B, Wichmann D, Emmerich P, Schmidt-Chanasit J, de Heer G, Kluge S (2014). A case of severe Ebola virus infection complicated by gram-negative septicemia. N Engl J Med.

[30] Qiu X, Wong G, Audet J, Bello A, Fernando L, Alimonti JB (2014). Reversion of advanced Ebola virus disease in nonhuman primates with ZMapp. Nature.

[31] WHO: WHO convenes industry leaders and key partners to discuss trials and production of Ebola vaccine [http://www.who.int/mediacentre/news/releases/2014/ebola-vaccines-production/en/]

[32] Wang C, Cao B, Liu QQ, Zou ZQ, Liang ZA, Gu L (2011). Oseltamivir compared with the Chinese traditional therapy maxingshigan-yinqiaosan in the treatment of H1N1 influenza: a randomized trial. Ann Intern Med.

[33] WHO Ebola Response Team (2014). Ebola virus disease in West Africa–the first 9 months of the epidemic and forward projections. N Engl J Med.

[34] Chen T, Ka-Kit Leung R, Liu R1, Chen F, Zhang X, Zhao J (2014). Risk of imported Ebola virus disease in China. Travel Med Infect Dis.

[35] Check Hayden E (2014). World struggles to stop Ebola. Nature.

